# First person – Maria Replogle

**DOI:** 10.1242/dmm.038117

**Published:** 2018-12-12

## Abstract

First Person is a series of interviews with the first authors of a selection of papers published in Disease Models & Mechanisms, helping early-career researchers promote themselves alongside their papers. Maria Replogle is first author on ‘ Establishment of a murine culture system for modeling the temporal progression of cranial and trunk neural crest cell differentiation’, published in DMM. Maria is a PhD student (dissertator) in the lab of Ava J. Udvadia at University of Wisconsin-Milwaukee, Milwaukee, USA, investigating the genetic and environmental factors that contribute to developmental birth defects and disorders, particularly those that impact the formation of the skeletal elements in the head.


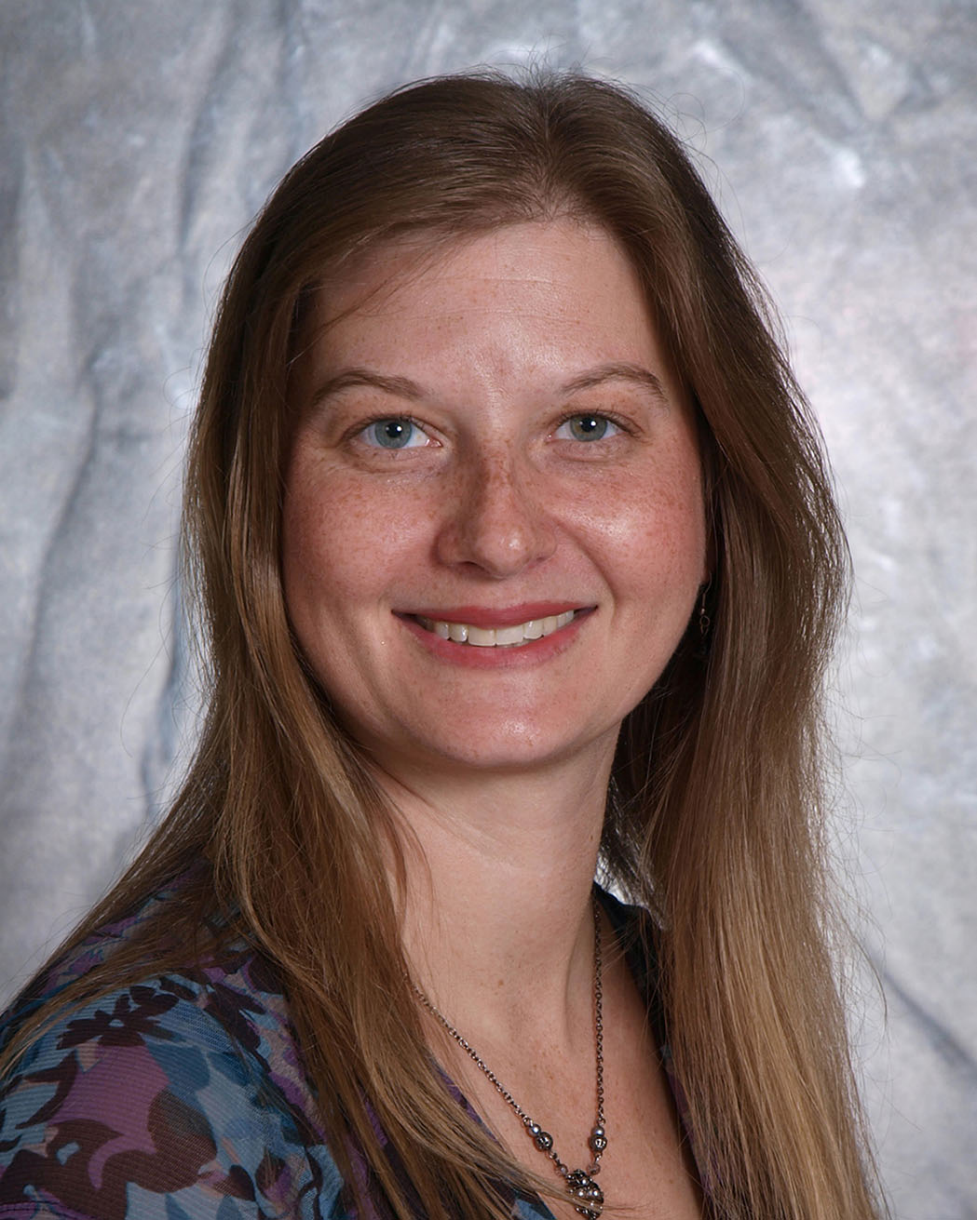


**Maria Replogle**

**How would you explain the main findings of your paper to non-scientific family and friends?**

The neural crest (NC) is a population of cells with the remarkable ability to produce many different cell types in a developing baby, ranging from pigment cells to neurons to cartilage and bone. Abnormal development of the NC can lead to a wide variety of birth defects and pediatric syndromes, many of which are characterized by facial deformities. Understanding the mechanisms that drive these cells to transition into a specific cell type, like the ones that produce cartilage in the head or sensory neurons in the limbs, is crucial to figuring out how genetic and environmental factors contribute to NC-related defects. One limitation we face is that the NC is a relatively small population of cells, only present during the early stages of embryonic development. This makes it difficult to conduct the experiments needed to determine the mechanisms that govern NC cell behavior. In our resource article, we present a reliable method for expanding and directing cell-type-specific transitions in both cranial and trunk NC cells isolated from embryonic mice. Significantly, we were able to identify highly reproducible benchmarks that track how these cells change over time as they form various cell types in culture, including neurons and cartilage-producing cells. In fact, many of the changes we observed in culture mimic certain attributes that these NC cells are known to exhibit during these same processes within the developing embryo. Having insight into the intermediate stages that the NC cells normally undergo as they form specific cell types greatly enhances our ability to determine how genetic mutation or environmental disruptions to these time-sensitive processes contributes to NC-related birth defects. In this light, our culture system not only provides a foundation for future investigations into the temporal aspect governing the formation of tissues that arise from the NC, but it also serves as a platform for screening drugs for developmental toxicity or therapeutic potential.

“[…] we were able to identify highly reproducible benchmarks that track how these [neural crest] cells change over time as they form various cell types in culture, including neurons and cartilage-producing cells.”

**What are the potential implications of these results for your field of research?**

Our culture system serves as a powerful tool for studying the molecular mechanisms that regulate NC cell growth and differentiation, both under normal and pathogenic conditions. One unique aspect of our culturing method is that we are able to isolate both cranial and trunk NC cells from the same embryos. This allows us to make direct, side-by-side comparison between the two cell populations *in vitro*. In some cases, we were able to observe subtle variations in the way these cells differentiated towards specific cell fates over time. Having the ability to detect such variations enables further investigation into the distinct regulatory mechanism underlying cranial and trunk NC-specific lineage acquisition. It also provides a platform to determine how differentiation in each cell population might be differentially impacted by genetic or environmental insult, which can be difficult to tease apart *in vivo*. Along these lines, we envision that future studies could utilize our *in vitro* methods to analyze NC cells isolated from mouse models of human NC-related disorders. Such analyses would help to distinguish the specific effect of genetic mutation on NC growth and differentiation, separately from a non-cell-autonomous environmental contribution, with focus on the impact at the level of single cell types and between derivatives of the two NC cell populations.

**Figure:**
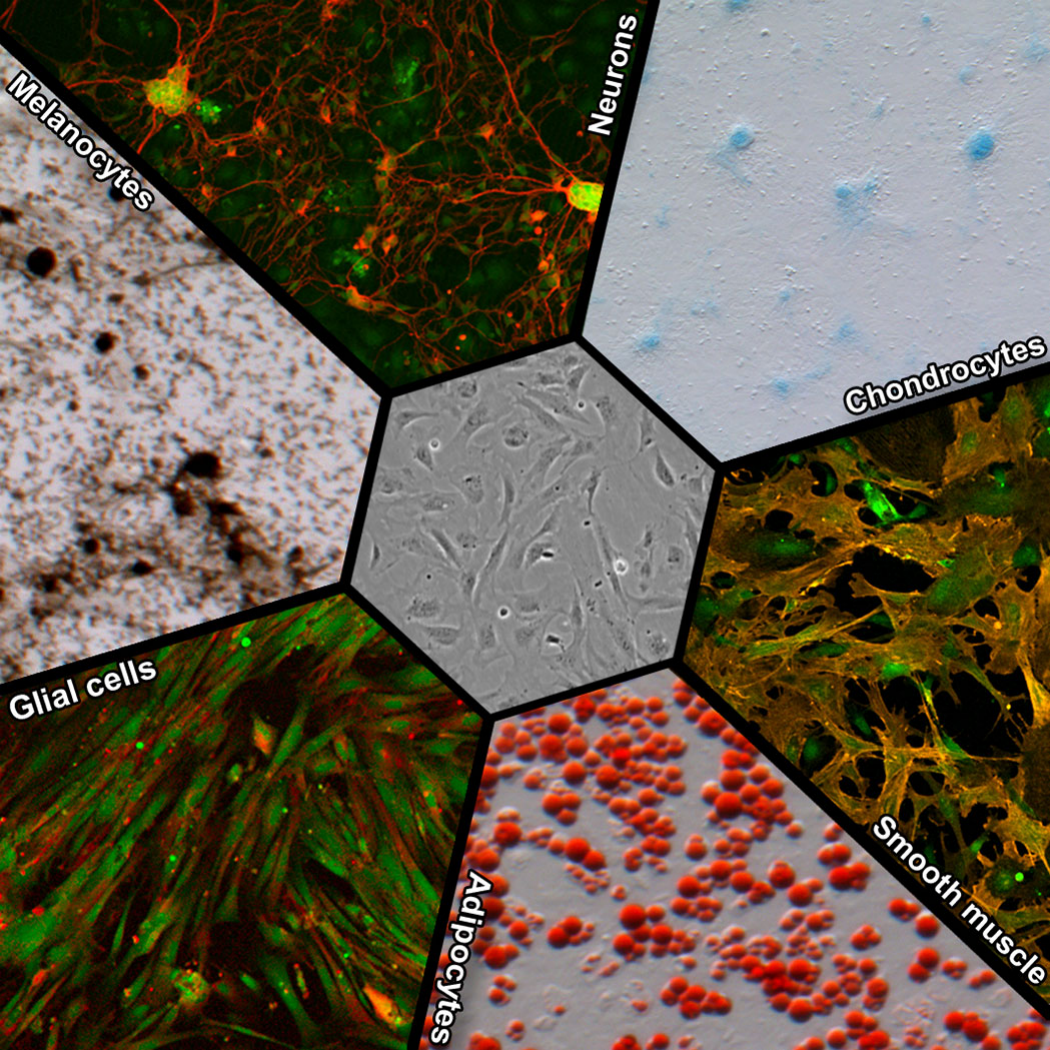
**Primary murine neural crest cells can give rise to both ectodermal and mesoectodermal derivatives in culture, including (clockwise from the top) neurons, chondrocytes, smooth muscle, adipocytes, glial cells and melanocytes.**

**What are the main advantages and drawbacks of the model system you have used as it relates to the disease you are investigating?**

*In vitro* models of the NC are beneficial for studying many aspects of NC cell development; however, it is often difficult to culture these cells in such a way that they can be maintained long-term. In our *in vitro* model system, primary cranial and trunk NC cells can be reliably isolated and expanded in culture for an extended period of time, all the while retaining their NC cell identity, capacity for self-renewal and ability to give rise to numerous NC derivatives. Notably, the cultured NC cells can be rapidly expanded into the millions within just the first few passages, yielding sufficient cell numbers to conduct genomic and proteomic analyses to investigate molecular function. One drawback to this model system is the necessity to maintain a mouse colony for continued isolation of the primary NC cells for culture and downstream analysis. However, this aspect also offers versatility as different mouse strains can be utilized to address different questions, allowing this culture system to be used in a variety of ways to investigate the mechanisms that regulate NC cell development both under normal conditions and in the context of NC-related defects. In addition, thorough characterization of the progression of differentiation *in vitro* provided insight into the temporal changes the cranial and trunk NC cells undergo as they transition towards a specific cell lineage. Establishing a chronological baseline for how these cells differentiate over time greatly increases our ability to determine how genetic manipulation or exposure to environmental compounds might speed up or slow down these processes, thus contributing to NC-related anomalies.

**What has surprised you the most while conducting your research?**

It was amazing to see that there are certain behaviors that many of the derivatives appear to share as they undergo differentiation in culture. For instance, the NC cells coalesce to form aggregations during both neuronal and chondrogenic differentiation, although there are differences in the extent of aggregation between the cranial and trunk NC. Having a better understanding of the mechanisms that drive these shared characteristics may shed light on the pleiotropic structural and functional defects often seen in patients with syndromes or disorders relating to abnormal NC cell development.

**Describe what you think is the most significant challenge impacting your research at this time and how will this be addressed over the next 10 years?**

Over the past few decades, we have learned considerably about the key players that contribute to many of the congenital anomalies resulting from disruptions in NC growth, migration or differentiation. However, the syndromes associated with NC-related defects, termed neurocristopathies, often have distinct – but sometimes overlapping – symptoms, each of which have the potential to exhibit varying prevalence and severity. This makes it difficult to correctly diagnose patients and implement appropriate treatment and therapy. While genetic screening is still the gold standard for diagnosis, a comprehensive list of candidate genes that contribute to the different neurocristopathies have not been fully identified. *In vitro* models of the NC can be particularly helpful for identifying putative candidate genes that can be used as markers for future diagnostic tools and the development of potential therapeutics. A related challenge to overcome would be to reduce the overall incidence of NC-related birth defects and neurocristopathies in the population. One step in addressing this issue requires more comprehensive ways to test personal use products, industrial compounds and therapeutic drugs for developmental toxicity. Current whole-animal testing methods are expensive and time consuming, especially considering the large number of compounds that need to be assessed. *In vitro* assays provide a platform for quickly screening through these compounds to prioritize the list for further testing in whole animals.

“[…] to address the big biological questions, you need to be a bit of a physicist, a chemist, a statistician and an engineer.”

**What changes do you think could improve the professional lives of early-career scientists?**

Making the transition from postdoctoral fellow to new faculty member can be quite daunting. This transition may be improved through implementation of training programs designed to prepare new faculty members for the challenges of setting up a new lab, new course design and increased professional responsibilities. I believe that, equipped with the right tools and with clearer expectations from the start, a scientist making this transition would be able to better organize their approach, allowing them to carve out more time to develop fundable ideas. I think it is also important for early-career scientists to expand their thinking when it comes to collaborative efforts. I believe that fostering relationships outside your own department to establish interdisciplinary collaborations should be encouraged more. Through my own experiences, I have come to realize that, sometimes in order to address the big biological questions, you need to be a bit of a physicist, a chemist, a statistician and an engineer. It is challenging to wear all these hats, especially as an early-career scientist, but it is not necessary to do it alone.

**What's next for you?**

Currently, we are using our culture system to investigate protein-protein and protein-gene interactions that regulate the timing of cranial NC-derived chondrogenic differentiation. We are also involved in an interdisciplinary collaboration using our culture system to determine the direct impact of nicotine on neuronal and chondrogenic differentiation. We expect that the findings of these studies will fundamentally advance our understanding of the mechanisms that contribute to craniofacial defects and disorders. My long-term goal is to pursue a career in academia. As a future faculty member at a university, I plan to further contribute to advances in the scientific and healthcare fields, as well as share my passion for science through teaching and mentoring the next generation of scientists.
